# Functions of CaPhm7 in the regulation of ion homeostasis, drug tolerance, filamentation and virulence in *Candida albicans*

**DOI:** 10.1186/s12866-018-1193-9

**Published:** 2018-06-04

**Authors:** Linghuo Jiang, Hongbo Pan

**Affiliations:** Laboratory for Yeast Molecular and Cell Biology, the Research Center of Fermentation Technology, School of Agricultural Engineering and Food Science, Shandong University of Technology, Zibo, 255000 China

**Keywords:** Anoctamin, TMEM16, Calcium permeable stress-gated cation channel, Phm7, CSC1, Ion

## Abstract

**Background:**

Calcium-permeable transient receptor potential (TRP) channels exist in eukaryotic cells from yeasts to animals and plants. and they act as sensors for various stresses. *Arabidopsis thaliana* calcium permeable stress-gated cation channel 1 (AtCSC1) was the first plant calcium-permeable TRP to be described and can be activated by hyperosmotic shock. *Candida albicans CaPHM7* is one of the sequence homologs of AtCSC1, but its function remains unknown.

**Results:**

We show here that CaPhm7 is localized to the plasma membrane in both the yeast and hyphal cells of *C. albicans*. *C. albicans* cells lacking *CaPHM7* are sensitive to SDS and ketoconazole but tolerant to rapamycin and zinc. In addition, deletion of *CaPHM7* leads to a filamentation defect, reduced colony growth and attenuated virulence in the mouse model of systemic infection.

**Conclusions:**

CaPhm7 is involved in the regulation of ion homeostasis, drug tolerance, filamentation and virulence in this important human fungal pathogen. CaPhm7 could be a potential target of antifungal drugs.

**Electronic supplementary material:**

The online version of this article (10.1186/s12866-018-1193-9) contains supplementary material, which is available to authorized users.

## Background

Calcium homeostasis and calcium signaling are highly conserved during evolution in eukaryotic cells [1, 2]. The cytosolic calcium concentration is maintained by calcium channels, pumps, exchangers and other regulators in the plasma membrane and intracellular organelle membranes of eukaryotic cells [2–4]. Elevation of the cytosolic calcium concentration is one of the earliest responses to stress in eukaryotic cells [5, 6]. Calcium-permeable transient receptor potential (TRP) channels exist in animals, plants and yeasts, and act as sensors for various stresses, including temperature, pH, osmolarity and nutrient availability [7–13]. The first calcium-permeable TRP, initially isolated from *Arabidopsis thaliana*, can be activated by hyperosmotic shock and, therefore, is named as calcium permeable stress-gated cation channel 1 (CSC1) [8]. AtCSC1 was also independently isolated through a genetic approach and named as reduced hyperosmolarity-induced [Ca^2+^]_i_ increase 1 (OSCA1) [7]. AtCSC1 and its *Saccharomyces cerevisiae* homolog *ScCSC1* also belong to the anoctamin/TMEM16 family [8].

The anoctamin/TMEM16 family members function in many physiological processes, including ion homeostasis and phospholipid scrambling [14–16]. TMEM16A (also known as ANO1) and TMEM16B (ANO2) form the Ca^2+^-activated Cl^−^ channel, which is important for transepithelial ion transport, olfaction, phototransduction, smooth muscle contraction, nociception, cell proliferation and control of neuronal excitability [14]. The Ca2^+^-activated Cl^−^ channel formed by CaCC and TMEM16F regulates phospholipid scramblase [15]. TMEM16F itself also functions as a phospholipid scramblase [16]. However, functions of other family members remain to be elucidated. Mutations in four anoctamin genes (ANO3, ANO5, ANO6, and ANO10) cause various genetic diseases. For example, mutations in the plasma membrane protein TMEM16F impair Ca^2+^-dependent externalization of phosphatidylserine in activated platelets, erythrocytes and lymphocytes, which leads to Scott syndrome, a bleeding disorder [15, 16]. In addition, a few other anoctamin-related diseases have been identified, such as ataxia and dystonia, persistent borrelia and mycobacteria infection, skeletal syndromes, gnathodiaphyseal dysplasia as well as cancers [17]. TMEM16C (ANO3) is associated with childhood mumps and rubella vaccination-related febrile seizures [18].

*Candida albicans* is the most common opportunistic human fungal pathogen, which causes life-threatening systemic infections in immunocompromised patients [19, 20]. To explore the biological functions of *C. albicans* homologs of CSC1 and other anoctamin/TMEM16 family members, using the amino acid sequence of AtCsc1 as a query, we blasted the *C. albicans* genome and revealed five AtCsc1 homologous proteins, CaPhm7, CaRsn1, CaSpo75, orf19.1823 (C1_06270W) and orf19.5932 (C3_04720C). CaPhm7 is a protein of 927 amino acids, which shares 21% (42%), 36% (59%), 26% (44%), 20% (41%) and 21% (42%) identities (similarities) with AtCsc1, CaRsn1, CaSpo75, C1_06270Wp and C3_04720Cp, respectively, in amino acid sequences (Additional file 1: Figure S1). In this study, we have characterized the biological functions of *CaPHM7* in *C. albicans*.

## Methods

### Strains, media, plasmids and primers

*C. albicans* strains and plasmids used in this study are listed in Tables 1 and 2, respectively. Primers used in this study are listed in Additional file 1: Table S1. *C. albicans* cells were maintained in YPD medium (2% glucose, 2% peptone, 1% yeast extract) or SD medium (0.67% yeast nitrogen base without amino acids, 2% glucose, and auxotrophic amino acids as needed) at 30 °C. Antifungal drugs were obtained from Sigma.

**Table 1:** Strains used in this study

Strain	Genotype	Source
CAI4	*ura3::λimm434/ura3::λimm434*	[38]
PHBCA57	CAI4 *PHM7/phm7::natR*	This study
PHBCA58	CAI4 *PHM7/phm7::frt*	This study
PHBCA62	CAI4 *phm7::frt/phm7::natR*	This study
XDCA06	CAI4/CIp10	[32]
PHBCA100	CAI4 *PHM7/phm7::frt*/CIp10	This study
PHBCA102	CAI4 *phm7::frt/phm7::frt*/CIp10	This study
PHBCA105	CAI4 *phm7::frt/phm7::frt*/CIp10-CaPHM7	This study
PHBCA107	CAI4 *PHM7/PHM7::GFP-URA3*	This study
PHBCA126	CAI4 *PHM7::GFP-URA3/phm7::natR*	This study

**Table 2:** Plasmids used in this study

Plasmid	Description	Source
CIp10	*C. albicans* integration vector with *URA3* marker	[30]
CIp10-CaPHM7	Full-length *CaPHM7* gene in CIp10	This study
pSFS2	The *SAT1* flipper cassette	[23]
pGFP-URA3	C-terminal GFP integration with *URA3* marker	[24]

### DNA manipulation

There is one *Pst*I site (CT**GCA**G), which contains a codon (bolded), encoding the A499 residue of CaPhm7, in both alleles A and B of the *CaPHM7* gene. However, due to the presence of a synonymous coding single nucleotide polymorphism (GC**A**→GC**T**) (cSNP) between alleles A and B of the *CaPHM7* gene encoding the A705 residue of CaPhm7, there is an additional *Pst*I site (CTGC**A**G) overlapping with this cSNP site in the allele A of *CaPHM7*. Therefore, using the primer pair pRS316-CaPHM7-F/CaPHM7 TY-UP-R, we first amplified the DNA fragment containing the 739-bp promoter and the upstream 1498-bp ORF region of *CaPHM7*, cut it with *Hind*III and *Pst*I, and cloned it into the CIp10 vector, yielding CIp10-CaPHM7UP. Second, using the primer pair CaPHM7 TY-DOWN-F/CaPHM7 TY-DOWN-R, from the allele B of *CaPHM7* we amplified a 1.58-kb DNA fragment containing the downstream 1307-bp ORF region and the 321-bp terminator region of *CaPHM7*, cut with *Pst*I, and clone the 1.58-kb DNA fragment into the *Pst*I site of CIp10-CaPHM7UP, generating CIp10-CaPHM7. These inserts were sequenced to ensure no mutation and correct orientation.

### Disruption of two alleles for *CaPHM7*

To delete the first allele of *CaPHM7* in the CAI4 background, we PCR amplified the *SAT1* flipper cassette with primers CaPHM7-NAT-UP and CaPHM7-NAT-DOWN from the plasmid pSFS2 [21–23], and transformed it into the CAI4 strain. The resulting PHBCA57 strain (*PHM7*/*phm7*::*natR*) was grown in YPD medium containing 25 μg ml^− 1^ nourseothricin for FLP-mediated excision of the *SAT1* cassette, which generated PHBCA58 strain (*PHM7*/*phm7*::*frt*). Similarly, we disrupted the second allele, which generated the PHBCA62 strain (*phm7::frt*/*phm7*::*natR*) (Additional file 1: Figure S2).

### Chromosomal tagging of CaPhm7 with green fluorescent protein (GFP)

To generate a C-terminal GFP-tagged CaPHM7-GFP fusion protein, from the plasmid pGFP-URA3 [24], we used the primers CaPHM7-GFP-UP and CaPHM7-GFP-DOWN to amplify the GFP-URA3 cassette flanked with 100-bp *CaPHM7* DNA fragments. This cassette was transformed into both the wild type CAI4 and the heterozygous mutant PHBCA57. Genotypes of the resulted *CaPHM7-GFP* strains PHBCA107 and PHBCA126 were verified by PCR with primer pairs CaPHM7-GFP-F/CaPHM7-GFP-R and GFP-R/CaURA3-F, respectively (Data not shown).

### Phenotype tests

Test strains were grown overnight in liquid medium at 30 °C, serially diluted by 10-fold and spotted sequentially onto plates as described previously [25, 26]. Plates were photographed after they were incubated for 2–3 days at 30 °C. Filamentation was assessed by inoculating cells with an appropriate dilution into various filamentation-induction liquid media, and cells were grown with shaking at 37 °C. For colony morphology observation 20 cells from each strain was plated onto YPD 10% serum, Lee’s and Spider medium [27, 28], and incubated for 5–7 days at 37 °C. These experiments were repeated at least three times.

### Growth curve assay

Cells of the wild type CAI4 + CIp10, the heterozygous mutant *PHM7/phm7* + CIp10, the homozygous mutant *phm7/phm7* + CIp10 and the revertant *phm7/phm7* + CIp10-CaPHM7 were cultured overnight in SD-URA medium at 30 °C, serially diluted for 10 times in fresh YPD medium and grown further for 10 h. Culture samples of each strain were taken every 2 h for OD_600nm_ measurement. Data were the average of three independent experiments and analyzed by GraphPad Prism software. *P* values of < 0.05 were considered to be significant.

### Virulence assays

Strains tested for virulence were integrated with the plasmid CIp10, so that *URA3* was expressed from the neutral *RPS1* locus [29, 30]. Phenotypes of all CIp10-integrated mutants were confirmed (Data not shown). Male BALB/c mice of 8 weeks old and 25–30 g (Geruisiwei Inc., Suzhou, China) were maintained in individually ventilated cages. Cell suspension at a concentration of 1 × 10^7^ cells ml^− 1^ in 0.9% (*w*/*v*) NaCl solution was prepared for each strain. Twelve mice for each strain were inoculated with 0.1 ml of cell suspension per mouse via a lateral tail vein. Mouse survival rates were monitored daily. If mice appeared to be curled up, hair fluffy, dull and not responsive to outside stimuli, they were sacrificed through cervical vertebra. Survival curves were generated according to the Kaplan–Meier method using the PRISM program (GraphPad Software) and compared using the log-rank test.

After 48 h of inoculation, two mice were randomly executed, and the colony forming unit (CFU) in their kidneys was analyzed on SD-URA plates as described [31, 32]. Stained sections of kidney tissues were examined for fungal infiltration. Mouse studies were carried out in accordance with the guidelines established by the Ethics Committee of Nantong University, China.

## Results

### Deletion of *CaPHM7* causes *C. albicans* cells sensitive to SDS and ketoconazole and tolerant to rapamycin and zinc ion

**Fig. 1:**
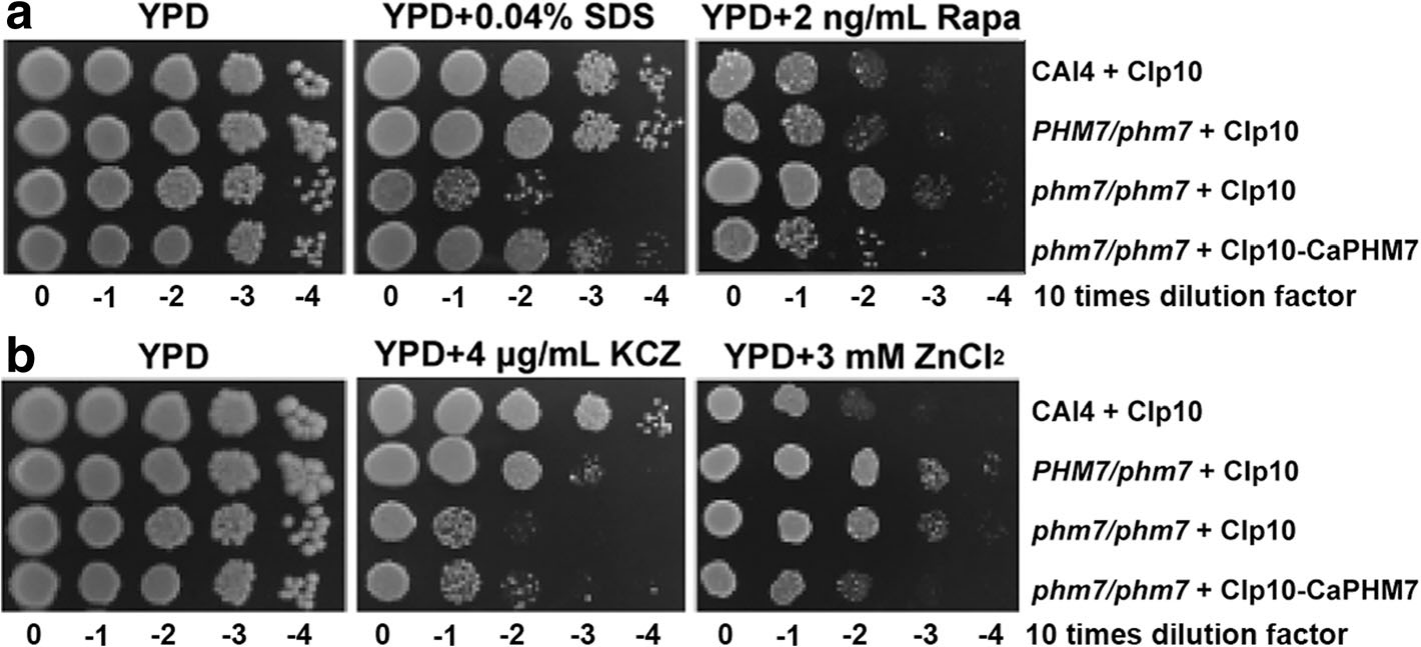
*C. albicans* cells lacking *CaPHM7* are sensitive to SDS and ketoconazole (KCZ) (**a**) and tolerant to rapamycin and zinc (**b**). The wild type, the heterozygous mutant and the homozygous mutant, integrated with the CIp10 vector or CIp10-CaPHM7, were grown overnight in SD-URA medium, serially diluted by 10 times and spotted onto YPD plates indicated. Plates were incubated at 30 °C for 48 h (**a**) or 72 h (**b**) before photos were taken. KCZ, ketoconazole; Rapa, rapamycin

Unlike AtCsc1 with nine TM domains, CaPhm7 has 11 TM domains (Additional file 1: Figure S1). However, CaPhm7 is highly conserved in ascomycetes including other human pathogenic *Candida* species, and shares 94% (98%), 42% (64%), 82% (91%), 43% (64%) and 46% (67%) identity (similarity), respectively, with *C. dubliniensis* CdPhm7, *C. glabrata* CgPhm7, *C. tropicalis* CtPhm7, *S. cerevisiae* ScPhm7 and *Schizosaccharomyces pombe* SpPhm7 (Additional file 1: Figure S3). To understand the biological functions of CaPhm7 in *C. albicans*, we disrupted the two alleles of *CaPHM7* (Table 1; Additional file 1: Figure S2). As compared to the wild type, the homozygous mutant was sensitive to SDS and tolerant to rapamycin (Fig. 1a). In contrast, both the heterozygous mutant and the homozygous mutant were sensitive to ketoconazole (KCZ) and tolerant to ZnCl_2_ (Fig. 1b). However, no alternation was observed in the sensitivity of these mutants to other agents such as calcium (0.6 M), manganese (1 mM), magnesium (10 mM), sodium (1.5 M), potassium (1.5 M), lithium and cadmium (100 μM) ions (Data not shown). The SDS-sensitive, rapamycin-tolerant and zinc-tolerant phenotypes of the homozygous mutant could be reversed by introducing one allele of the *CaPHM7* gene (Fig. 1). However, it should be noted that reintroduction of *CaPHM7* only partially restored growth of the homozygous mutant on ketoconazole. On zinc, the revertant (one copy of *CaPHM7*) restored the wild type phenotype, but the heterozygous mutant grew at levels similar to the homozygous mutant (Fig. 1). Furthermore, the colonies of both the homozygous mutant and the revertant were slightly smaller than those of the wild type on solid YPD plates (Fig. 1). However, the growth rates of both the homozygous mutant and the revertant were not significantly different from the wild type (Additional file 1: Figure S4).

### CaPhm7 is localized to the plasma membrane

**Fig. 2:**
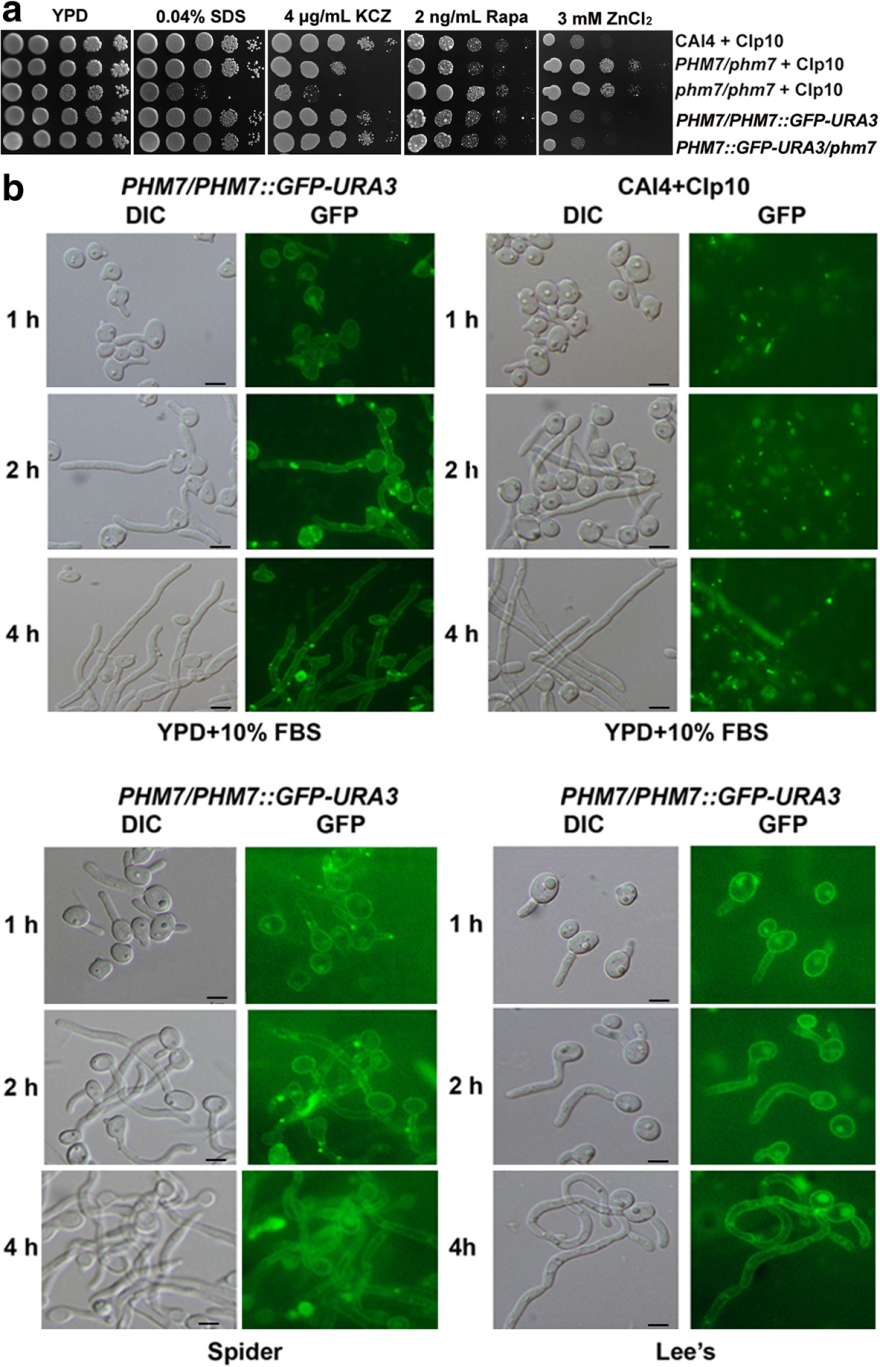
Subcellular localization and mouse virulence assay for CaPhm7. **a** Functional assay of the *CaPHM7-GFP* allele. The wild type CAI4 (XDCA06), the heterozygous mutant PHBCA100, the homozygous mutant PHBCA 102, as well as PHBCA107 and PHBCA126, carrying the *CaPHM7-GFP* allele, were grown overnight in SD-URA medium, serially diluted by 10 times and spotted onto YPD plates indicated. **b** Visualization of GFP signals within PHBCA107 cells of log-phase yeast-form and filamentous form induced in YPD + 10% FBS (top left), Spider (bottom left) or Lee’s (bottom right) media at 1000 × magnification on a Nikon ECLIPSE 80i microscope. Visualization of the wild type CAI4 cells (top right) as a control for hyphal morphology and green auto-fluorescence. DIC, differential interference contrast. The scale bars represent 5 μM

To examine the subcellular localization of CaPhm7 proteins, we integrated the GFP tag at the C-terminus of the *CaPHM7* allele in the wild type CAI4 and the heterozygous mutant, which generated PHBCA107 and PHBCA126 strains, respectively. Both showed similar phenotypes to the wild type CAI4 on plates containing SDS, ketoconazole, rapamycin or ZnCl2 (Fig. 2a), indicating the *CaPHM7-GFP* allele is functional. Western blot analysis indicated that the CaPHM7-GFP expressed as a protein of 126 kDa, as expected, in cells of both PHBCA107 and PHBCA126 (Additional file 1: Figure S5). This CaPHM7-GFP protein was localized to the plasma membrane in both the log-phase yeast-form cells and the filamentous cells of the PHBCA107 strain induced in YPD + 10% FBS, Spider or Lee’s media (Fig. 2b). Taken together, these data indicate that CaPhm7 is a plasma membrane protein.

### Deletion of *CaPHM7* leads to a defect in filamentation, reduced colony growth and attenuated virulence

**Fig. 3:**
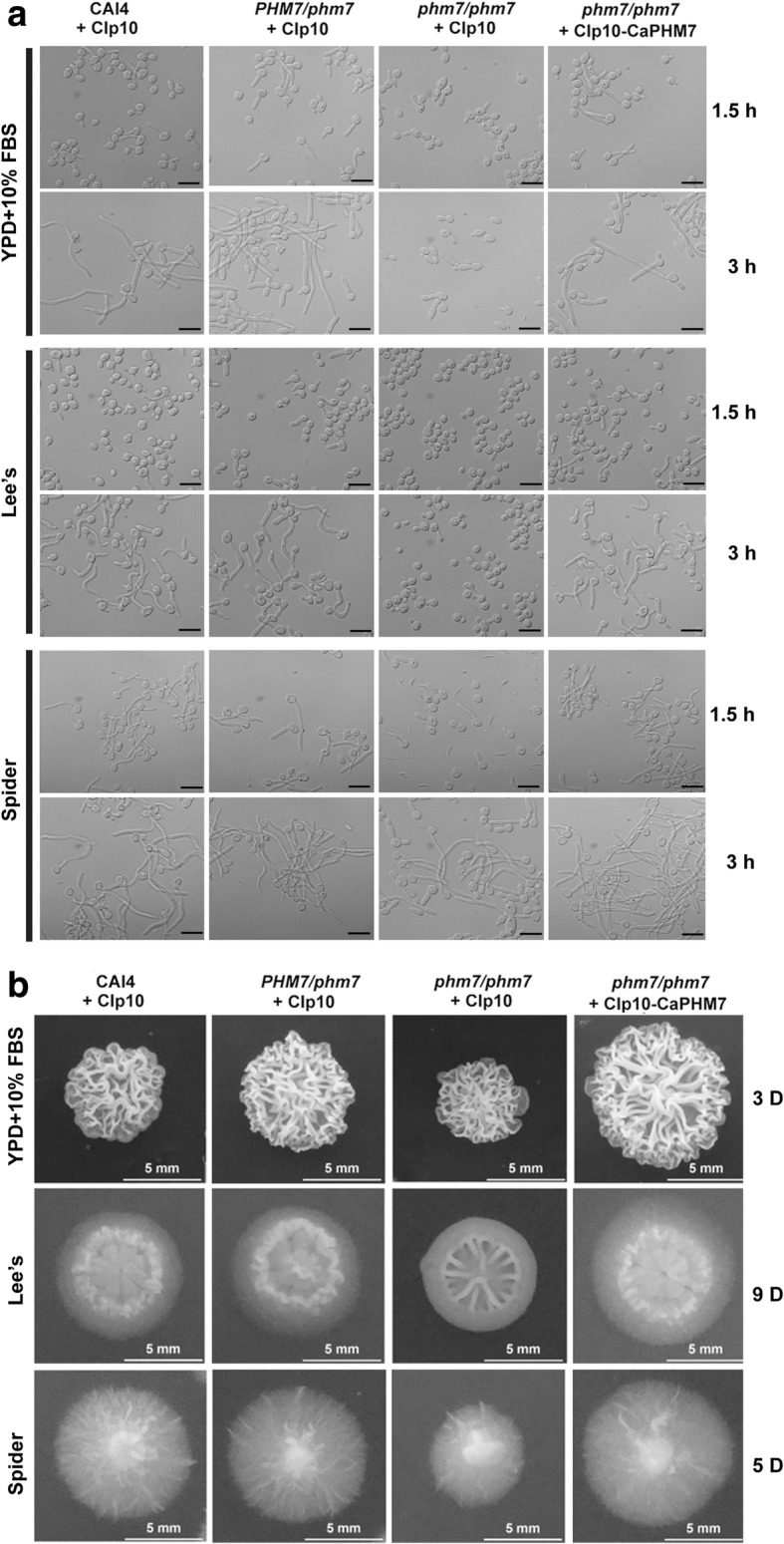
Functions of *CaPHM7* in filamentation and colony formation. The wild type CAI4, the heterozygous mutant and the homozygous mutant, carrying the CIp10 vector or CIp10-CaPHM7, were grown overnight in SD-URA medium, and inoculated to liquid YPD containing 10% fetal bovine serum (FBS), Lee’s or Spider media for filamentation assay (**a**) or onto their solid plates for colony morphology oberservation (**b**). Incubation was carried out at 37 °C. The scale bar in (**a**) represents 10 μM, while the scale bar in (**b**) represents 5 mm

Hyphal development is related to the virulence of *C. albicans* cells [20, 33]. To examine the function of *CaPHM7* in hyphal development, we performed filamentous growth assays. In liquid YPD plus 10% FBS, Lee’s or Spider media, the homozygous mutant showed a defect in filamentation (Fig. 3a). On these solid media, comparing to the wide type and the heterozygous mutant, the homozygous mutant showed reduced colony sizes on solid YPD plus 10% FBS, Lee’s and Spider media (Fig. 3b). These defects of the homozygous mutant could be complemented by reintroduction of a single copy of the *CaPHM7* allele (Fig. 3).

**Fig. 4:**
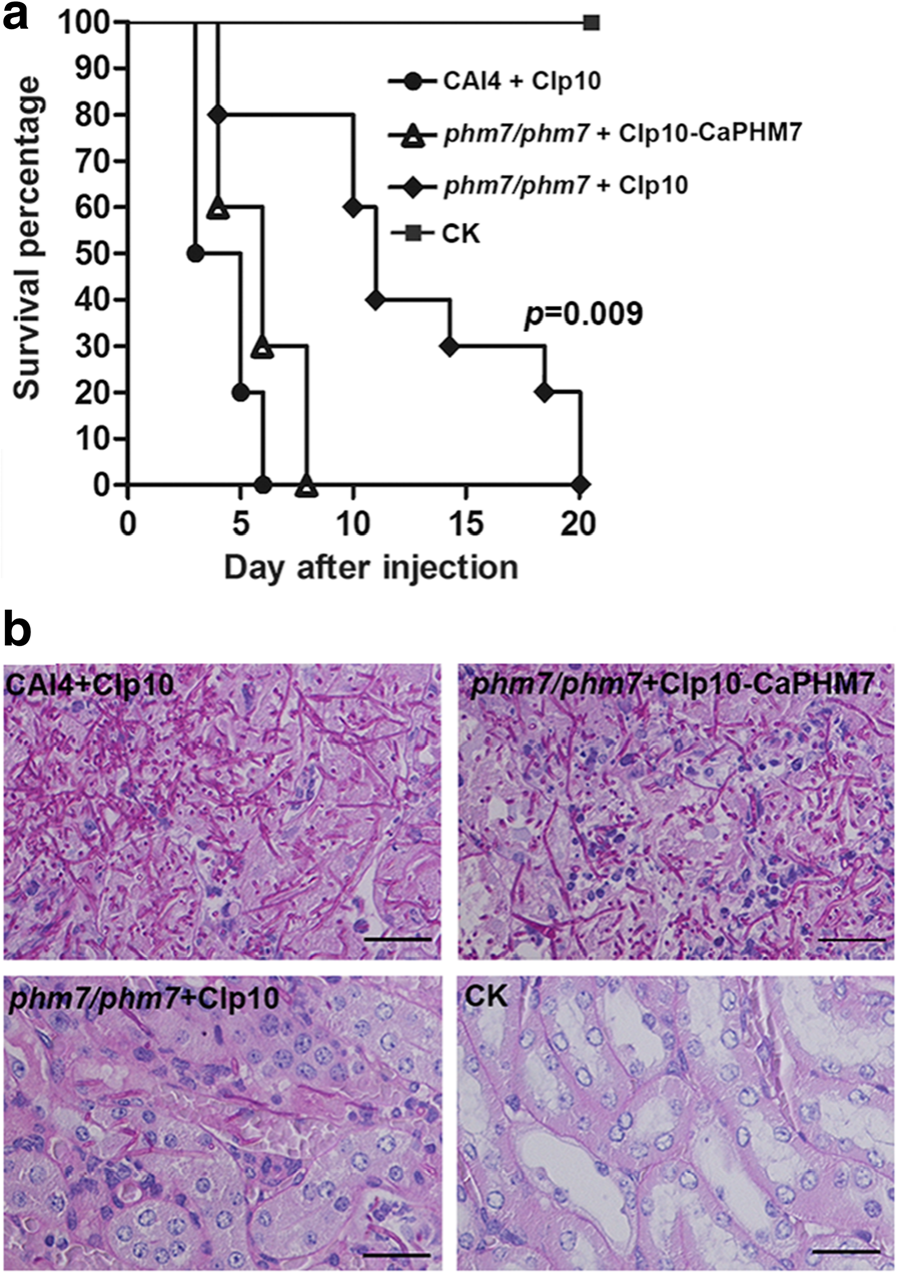
Virulence assay. **a** survival rates of mice (*n* = 10) infected with the wide type CAI4, the homozygous *phm7/phm7* mutant and its complemented strain. Mice were checked every day for morbidity, and survival was monitored for 30 days. The *p* value (0.009) of the log-rank test indicates that the survival curves between the wild-type CAI4 and the *phm7/phm7* mutant is significantly different. **b** histopathological examination of kidney tissues of moribund mice infected with the wild-type, the homozygous mutant and its complemented strain. Saline buffer was injected as negative control (CK). Infected kidney tissues from mice were stained with periodic acid-Schiff’s reagent. Representatives of five kidney cross-sections from two mice per strain were photographed with 40× lenses. The hyphal cells are highlighted by arrows. The scale bars represent 20 μM

To determine the role of *CaPHM7* in *C. albicans* virulence, we infected mice intravenously with 1 × 10^6^ cells of the wild-type parental control strain and the homozygous mutant. Two mice were randomly selected from each group and sacrificed after 48 h to make sure that mice had been infected, with the CFUs/g reaching 1.02 × 10^7^ (*n* = 2) in wet kidney tissue of the homozygous mutant, 1.56 × 10^7^ (*n* = 2) in that of the wild-type and 1.38 × 10^7^ (*n* = 2) in that of the complemented strain. By Day 6, none of the mice injected with the wild type survived, while 30% and 80% of the mice inoculated with the complemented strain and the homozygous mutant, respectively, survived. By Day 8, none of the mice inoculated with the complemented strain survived, while the mice injected with the homozygous mutant survived until Day 20 (Fig. 4a). As a control, no hyphal or yeast cells of *C. albicans* was observed in the kidney tissues of mice inoculated with saline as a control (Fig. 4b). Compared to kidney tissues of mice infected with the wild type and the complemented strain, those infected with the homozygous mutant were less infiltrated by hyphal filaments (Fig. 4b). Taken together, these results suggest that deletion of *CaPHM7* attenuates the virulence of *C. albicans* cells in this systemic infection model.

## Discussion

In this study, we have shown that the *C. albicans* homolog of AtCSC1, CaPhm7, is localized in the plasma membrane of both yeast-form and filamentous cells. *C. albicans* cells lacking *CaPHM7* are sensitive to membrane-disturbing agents, SDS and ketoconazole. AtCSC1 and ScCSC1 ion channels are permeable to various cations, including Ca^2+^, K^+^ and Na^+^ and can be activated by hyperosmotic shock [8]. However, lack of *CaPHM7* leads to a tolerance to Zn^2+^, but does not affect the sensitivity of *C. albicans* cells to other ions tested, including Ca^2+^, K^+^, Na^+^, Mg^2+^, Mn^2+^, Cd^2+^, and Li^+^. *C. albicans* cells lacking *CaPHM7* are also not sensitive to osmotic stresses such as 1 M mannitol and 4 M KCl. Therefore, whether CaPhm7 is a calcium permeable stress-gated cation channel in the plasma membrane remains to be determined. It is also interesting to note that haploinsufficiency occurs to the deletion of one of the *CaPHM7* alleles in the response to ketoconazole and zinc ion, but not to SDS and rapamycin (Fig. 1b). Among anoctamins, ANO10 shows the highest degree of homology to ScIst2, with a 36% similarity and a 17% identity in their amino acid sequences [17]. CaPhm7 shares 18% (34%) and 14% (30%) identity (similarity) with HsAno10 and ScPhm7, respectively, in their amino acid sequences (Additional file 1: Figure S6). This suggests that CaPhm7 might be a member of the anoctamin/TMEM16 family member.

In addition, we have shown that *C. albicans* cells lacking *CaPHM7* are tolerant to rapamycin, the specific inhibitor of the target of rapamycin (TOR) kinase, which indicates that the TOR kinase activity might be inhibited in *C. albicans* cells lacking *CaPHM7*. This is consistent with our observations that deletion of *CaPHM7* leads to a reduced colony growth, which might be related to a reduced activity for the central controller of cell growth, TOR [34].

The *S. cerevisiae* homolog of *CaPHM7*, *ScPHM7* has initially been identified to be the phosphate metabolism gene 7 and, together with other 21 genes, is regulated by the PHO regulatory pathway in *S. cerevisiae* [35]. However, the function of *ScPHM7* in phosphate metabolism is not defined up to now. ScPhm7 shares a 43% identity and 64% similarity, respectively, with CaPhm7 in amino acid sequence (Additional file 1: Figure S1). Our results on *CaPHM7* suggests that ScPhm7 might be a plasma membrane CSC ion channel that links the extracellular phosphate availability to the intracellular calcium signaling pathway to regulate phosphate metabolism. Recent studies have indeed linked the calcium signaling pathway to phosphate metabolism. Both the *PHO4* gene, encoding the transcription factor of the PHO pathway, and the *PHO89* gene, encoding the high-affinity inorganic phosphate (Pi) transporter, are transcriptionally regulated by the calcium/calcineurin signaling in *S. cerevisiae* [36]. Calcineurin is also involved in the regulation of the PHO pathway in *Aspergillus fumigatus* [37]. Therefore, like AtScs1 as an osmolarity sensor in the plant *A. thaliana* [7, 8], ScPhm7 might function as a sensor for extracellular phosphate levels in *S. cerevisiae*.

In this study, we have observed that the homozygous mutant for *CaPHM7* shows a filamentation defect, which might contribute to its reduced colony growth and attenuated virulence in the mouse model of systemic infection. Similar to the functions of other members of the anoctamin/TMEM16 family as sensors for various stresses including nutrient availability [7–13], the plasma membrane protein CaPhm7 might function to regulate zinc availability for *C. albicans* cells in media or within the host environment and thereby affect their filamentation and virulence.

## Conclusion

In this study, we have demonstrated that CaPhm7 is localized to the plasma membrane in both yeast-form and filamentous cells of *C. albicans*. CaPhm7 regulates ion homeostasis, drug tolerance, filamentation and virulence in this important human fungal pathogen. *C. albicans* cells lacking *CaPHM7* have a defect in filamentation and attenuated virulence in the systemic mouse model. Therefore, CaPhm7 is a potential target for antifungal development.

## Additional file

Additional file 1: **Figure S1.** Amino acid sequence comparison. Transmembrane domain (TM) regions of AtCsc1 and CaPhm7 are underlined, and TM names of AtCsc1 (blue) and CaPhm7 (red) are indicated. TMs were predicted with SMART (http://smart.embl-heidelberg.de/). Identical amino acid residues are indicated in yellow in color, and similar amino acid residues are indicated in light blue and green. **Figure S2.** Disruption of *CaPHM7*. (A) Disruption strategy of the two alleles. PCR confirmation of genotypes of the homozygous mutant PHBCA62 with primer pairs CaPHM7-ORF-UP/DOWN (B), CaPHM7-DF/DR (C) as well as CaPHM7-DF/NAT-R (middle lane) and CaPHM7-DR/NAT-F (last lane) (D). **Figure S3.** TMs of AtCsc1, ScPhm7 and CaPhm7 as well as their amino acid sequence comparison. Identical amino acid residues are indicated in yellow in color, and similar amino acid residues are indicated in light blue and green. **Figure S4.** Growth assay. Cells were cultured in SD-URA medium at 30 °C overnight, diluted for 10 times in fresh YPD medium and grown further for indicated hours. Data were the average of three independent experiments. Growth rates of the heterozygous mutant, the homozygous mutant and the revertant were not significantly different from the wild type. **Figure S5.** Western blot analysis. The CAI4 control, PHBCA107 and PHBCA126 cells were grown to log-phase and collected for total protein extraction. Western blot analysis was carried out with the monoclonal anti-GFP antibody. The CaPHM7-GFP protein of 126 kDa was indicated. **Figure S6.** Amino acid sequence comparison. CaPhm7 shares 18% (34%) and 14% (30%) identity (similarity) with human HsAno10 and *S. cerevisiae* ScIst2, respectively. Identical amino acid residues are indicated in yellow in color, and similar amino acid residues are indicated in light blue and green. **Table S1.** Primers used in this study. (PDF 5687 kb)

## Abbreviations

CSC: Calcium permeable stress-gated cation channel
TRP: Calcium-permeable transient receptor potential

## References

[1] Plattner H, Verkhratsky A (2015). The ancient roots of calcium signalling evolutionary tree. Cell Calcium.

[2] Cui J (2009). Calcium homeostasis and signaling in yeast cells and cardiac myocytes. FEMS Yeast Res.

[3] Zhao Y (2016). The plasma membrane protein Rch1 is a negative regulator of cytosolic calcium homeostasis and positively regulated by the calcium/calcineurin signalling pathway in budding yeast. Eur J Cell Biol.

[4] Jiang L (2012). The *Candida albicans* plasma membrane protein Rch1p, a member of the vertebrate SLC10 carrier family, is a novel regulator of cytosolic Ca^2+^ homoeostasis. Biochem J.

[5] Peiter E (2016). The ever-closer union of signals: propagating waves of calcium and ROS are inextricably linked. Plant Physiol.

[6] Swarbreck SM, Colaço R, Davies JM (2013). Plant calcium-permeable channels. Plant Physiol.

[7] Yuan F (2014). OSCA1 mediates osmotic-stress-evoked Ca^2+^ increases vital for osmosensing in *Arabidopsis*. Nature.

[8] Hou C (2014). DUF221 proteins are a family of osmosensitive calcium permeable cation channels conserved across eukaryotes. Cell Res.

[9] Chang Y (2010). Properties of the intracellular transient receptor potential (TRP) channel in yeast, Yvc1. FEBS Lett.

[10] Venkatachalam K, Montell C (2007). Annu Rev Biochem.

[11] Bonilla M, Cunningham KW (2002). Calcium release and influx in yeast: TRPC and VGCC rule another kingdom. Sci STKE.

[12] Denis V, Cyert MS (2002). Internal Ca^2+^ release in yeast is triggered by hypertonic shock and mediated by a TRP channel homologue. J Cell Biol.

[13] Harteneck C, Plant TD, Schultz G (2000). From worm to man: three subfamilies of TRP channels. Trends Neurosci.

[14] Picollo A, Malvezzi M, Accardi A (2015). TMEM16 proteins: unknown structure and confusing functions. J Mol Biol.

[15] Pedemonte N, Galietta LJ (2014). Structure and function of TMEM16 proteins (aoctamins). Physiol Rev.

[16] Williamson P (2016). Phospholipid Scramblases. Lipid Insights.

[17] Kunzelmann K (2016). Modulating Ca^2+^ signals: a common theme for TMEM16, Ist2, and TMC. Pflugers Arch.

[18] Feenstra B (2014). Common variants associated with general and MMR vaccine-related febrile seizures. Nat Genet.

[19] Calderone RA, Fonzi WA (2001). Virulence factors of *Candida albicans*. Trends Microbiol.

[20] Mayer FL, Wilson D, Hube B (2013). *Candida albicans* pathogenicity mechanisms. Virulence.

[21] Feng J, et al. CaTip41 regulates protein phosphatase 2A activity, CaRad53 deactivation and the recovery of DNA damage-induced filamentation to yeast form in *Candida albicans*. FEMS Yeast Res. 2016;16 10.1093/femsyr/fow009. 10.1093/femsyr/fow009 26851402

[22] Wang Y, et al. Genetic interactions between the Golgi Ca^2+^/H^+^ exchanger Gdt1 and the plasma membrane calcium channel Cch1/Mid1 in the regulation of calcium homeostasis, stress response and virulence in *Candida albicans*. FEMS Yeast Res. 2015;15(7). 10.1093/femsyr/fov069 26208803

[23] Reuss O, Vik A, Kolter R, Morschhauser J (2004). The *SAT1* flipper, an optimized tool for gene disruption in *Candida albicans*. Gene.

[24] Bachewich C, Nantel A, Whiteway M (2005). Cell cycle arrest during S or M phase generates polarized growth via distinct signals in *Candida albicans*. Mol Microbiol.

[25] Liu W (2010). The protein kinase CaSch9p is required for the cell growth, filamentation and virulence in the human fungal pathogen *Candida albicans*. FEMS Yeast Res.

[26] Jiang L, et al. The putative ABC transporter encoded by the orf19.4531 plays a role in the sensitivity of *Candida albicans* cells to azole antifungal drugs. FEMS Yeast Res. 2016;16 10.1093/femsyr/fow024. 10.1093/femsyr/fow024 26975389

[27] Lee K, Buckley HR, Campbell CC (1975). An amino acid liquid synthetic medium for the development of mycelial and yeast forms of *Candida albicans*. Sabouraudia.

[28] Gimeno CJ, Ljungdahl PO, Styles CA, Fink GR (1992). Unipolar cell divisions in the yeast *Saccharomyces cerevisiae* lead to filamentous growth: regulation by starvation and RAS. Cell.

[29] Murad AM (2000). CIp10, an efficient and convenient integrating vector for *Candida albicans*. Yeast.

[30] Brand A (2004). Ectopic expression of *URA3* can influence the virulence phenotypes and proteome of *Candida albicans* but can be overcome by targeted reintegration of *URA3* at the *RPS10* locus. Eukaryot Cell.

[31] Li X (2010). The MAP kinase-activated protein kinase Rck2p regulates cellular responses to cell wall stresses, filamentation and virulence in the human fungal pathogen *Candida albicans*. FEMS Yeast Res.

[32] Xu D (2015). Genetic interactions between Rch1 and the high-affinity calcium influx system Cch1/Mid1/Ecm7 in the regulation of calcium homeostasis, drug tolerance, hyphal development and virulence in Candida albicans. FEMS Yeast Res.

[33] Gow NA (2011). *Candida albicans* morphogenesis and host defence: discriminating invasion from colonization. Nat Rev Microbiol.

[34] Heitman J (2015). On the discovery of TOR as the target of rapamycin. PLoS Pathog.

[35] Ogawa N, DeRisi J, Brown PO (2000). New components of a system for phosphate accumulation and polyphosphate metabolism in *Saccharomyces cerevisiae* revealed by genomic expression analysis. Mol Biol Cell.

[36] Serra-Cardona A (2014). Coregulated expression of the Na+/phosphate Pho89 transporter and Ena1 Na+-ATPase allows their functional coupling under high-pH stress. Mol Cell Biol.

[37] da Silva Ferreira ME (2007). Functional characterization of the *Aspergillus fumigatus* calcineurin. Fungal Genet Biol.

[38] Fonzi WA, Irwin MY (1993). Isogenic strain construction and gene mapping in *Candida albicans*. Genetics.

