# Supplementary material for: Functions of CaPhm7 in the regulation of ion homeostasis, drug tolerance, filamentation and virulence in Candida albicans
Source: BMC Microbiol. 2018 Jun 4;18:49. doi: 10.1186/s12866-018-1193-9 (PMC5987382; doi:10.1186/s12866-018-1193-9)
Supplement: Supplementary file 1 — Figure S1. Amino acid sequence comparison. Transmembrane domain (TM) regions of AtCsc1 and CaPhm7 are underlined, and TM names of AtCsc1 (blue) and CaPhm7 (red) are indicated. TMs were predicted with SMART (http://smart.embl-heidelberg.de/). Identical amino acid residues are indicated in yellow in color, and similar amino acid residues are indicated in light blue and green. Figure S2. Disruption of CaPHM7. (A) Disruption strategy of the two alleles. PCR confirmation of genotypes of the homozygous mutant PHBCA62 with primer pairs CaPHM7-ORF-UP/DOWN (B), CaPHM7-DF/DR (C) as well as CaPHM7-DF/NAT-R (middle lane) and CaPHM7-DR/NAT-F (last lane) (D). Figure S3. TMs of AtCsc1, ScPhm7 and CaPhm7 as well as their amino acid sequence comparison. Identical amino acid residues are indicated in yellow in color, and similar amino acid residues are indicated in light blue and green. Figure S4. Growth assay. Cells were cultured in SD-URA medium at 30 °C overnight, diluted for 10 times in fresh YPD medium and grown further for indicated hours. Data were the average of three independent experiments. Growth rates of the heterozygous mutant, the homozygous mutant and the revertant were not significantly different from the wild type. Figure S5. Western blot analysis. The CAI4 control, PHBCA107 and PHBCA126 cells were grown to log-phase and collected for total protein extraction. Western blot analysis was carried out with the monoclonal anti-GFP antibody. The CaPHM7-GFP protein of 126 kDa was indicated. Figure S6. Amino acid sequence comparison. CaPhm7 shares 18% (34%) and 14% (30%) identity (similarity) with human HsAno10 and S. cerevisiae ScIst2, respectively. Identical amino acid residues are indicated in yellow in color, and similar amino acid residues are indicated in light blue and green. Table S1. Primers used in this study. (PDF 5687 kb) [file 12866_2018_1193_MOESM1_ESM.pdf]

```
                                                              TM1
AtCsc1      1 -------------MATLQ------------------DIGVSAGINILSAF
CaPhm7      1 -------------MAGSSIPQSSNS--------------------SVSQF
CaRsn1      1 ----------------MAVAPSSVS---------------------TSTI
CaSpo75     1 MSSITVEQYDYQTLTKSYMDIFNSQKFAQFLNTTQTGFAHSSSGIEFSVL
C1_06270Wp  1 ----MLLLDILSIGWQIIIQTGTNGLGEDYTNGDNDGKKPDPIHSVGPQR
C3_04720Cp  1 ------MIDNIINNLQIILQQNDDNFTSPHD---------DVIYRPHSAR

                     TM1
AtCsc1     20 VFFIIFAVLRLQPFNDRVYFSKWYLKGLRSSPARGGAF-----AQRFVNL
CaPhm7     18 LSTLIPTLVVSVVFLLAFIVIHSKQRRVYEPRAVVESLP-------DDLR
CaRsn1     14 ISTLLANLALFGVFVGCFIVLRLKFKRIYSPKSSFDLVP-------EEKK
CaSpo75    51 MKTLFTSLCFCTIQLTLFCLLRPVFNYLYQPRCFCVPIN---------ER
C1_06270Wp 47 VFTTQLVLTLIAGMFSFLLFCMLRFKWPHIYAVRTLRQ----PRNNSHIL
C3_04720Cp 36 VARYQVIIASTLGLTALLLFSILRLKYPKIYVANFNHLNFSLHSTSRRNL

                                                         TM2  TM2
AtCsc1     65 DFRSYMKFLNWMPEALKMPEPELIDHAGLDSVVYLRIYWLGLKIFTPIAV
CaPhm7     61 TETAPKGPFSWLTYLLAKPRTFYIQYAGTDGYFFLRFLFEFFCVCVLGAV
CaRsn1     57 PEPLPKDPFRWIFILLTKPDSFFLQQAGLDGLVFLRYIKT-FGTLFLCAL
CaSpo75    92 METLPREFFKWIVPTLKCSIN-TYLSLGLDAYFFIRFISVLSLFFLFIGT
C1_06270Wp 93 RPLPNN-LFGWIKVVYKITDEEIIACSGLDTFVYLRFFKMGIKIFLILSI
C3_04720Cp 86 PELPSNSLFGWIPTVYKITEQEILEHAGLDAVVFLEFFKMCIRIISICLV

AtCsc1    115 LAWAVLVPVNWTNNT------LEMAKQLRNVTSSDIDKLSVSN-------
CaPhm7    111 ITWPILFPVNATNGNNNTPGSNVKGFDILTFAN-----------------
CaRsn1    106 LMYIILLPVNATNGN------HNKGFDQLSIAN-----------------
CaSpo75   141 LNMVILIPINYTGSS---TEYTAFGLDKLSLSN-----------------
C1_06270Wp142 SAIFVLSPIRYYFTG------NYDKENIMTKPNQPPDIN-----------
C3_04720Cp136 FAIIIISPIRYKFTG------RVDEDYPDDDSDNDDDDGSNNNGTTIIKH

                                             TM3 TM3
AtCsc1    152 --------------IPEYSMRFWTHIVMAYAFTIWTCYVLMKEYETIANM
CaPhm7    144 ---------------IKDKWRTFAHVFLSWILFGAVIFLIYRELVYYVTY
CaRsn1    133 ---------------VKHPRRYYAHVLMGLVFNGIVIFVIYRELFFYNSL
CaSpo75   171 -------------IATTNVSRLNAHFLMGLITIGFFHWLIVYEFQSYVII
C1_06270Wp175 ---------------YDFPSFYWVYPIFTYVFSIVVFYYLFEFTTTILRT
C3_04720Cp180 IVSAGISVASKNNDGEQYQQFLWLYTIFTYVFTFVTVYFLFKQTNRIISM

AtCsc1    188 RLQFVASE--ARRPDQFTVLVRNVPPDADES--------VSELVEHFFLV
CaPhm7    179 RHALQTTPLYDSLLSSRTLLLTEISTEYLQDDKLR----GYFPTATNIWY
CaRsn1    168 KNAVLSSPKYAKKLSCRTVLFQGVPDSLLDEKQAF----KIFNGVKRVYV
CaSpo75   208 RQSYLLSQPHKDSVMAKTLLISNVPPYLQNHEVLKTIFQVVPGGIKDIWD
C1_06270Wp210 RQKYLASQ--SS-ITDRTIKLDGIPKRLLQ----------REKLKKFIED
C3_04720Cp230 RQKYLGSQ--NS-VTDRTVKISGIPGSLRD----------EVALARHIDR

AtCsc1    228 NHPDHYLTHQVVCNANKLADLVKKKKKLQNWLDYYQLKYARNN-------
CaPhm7    225 SRDYKKLQKQVKERTKLANKYEGTLNKVLTKAVKLRNKCLKKSK------
CaRsn1    214 ARTARELEYKVEKRAAMVTKLENAENKLMKMAVKSKLKADKKG-------
CaSpo75   258 INEFEIIDHQVEIAQDALHYLEKSQVLGLKKYYHKKAQWCGSSVG-----
C1_06270Wp247 LGIGKVLDVKLIYNWTPLEDLLHKRQELMNNLECIYTSMYKMD-------
C3_04720Cp267 LNIGEVDSVLIVKEWQNLNKLFKRRRRIVRKLEESWVEYFEKNGITNKSD

AtCsc1    271 --------------SQ-RIMVKLGFLGLWG--------------------
CaPhm7    269 -PAPEPQDDIDKYLKDGKKRPTHKLKFLIG--------------------
CaRsn1    257 -IILEPVDEISSYVSE-KKRPKMKVGGFFS--------------------
CaSpo75   303 -------DSVEEIKEFIETHEIYFYPPLYSG-------------------
C1_06270Wp290 ---------IDIYNQH-EVPAVN---PIWS-------------------E
C3_04720Cp317 LISLHPQVGESYRFSN-RYTDDAEESPDWGSQNSNSAQASIIDQDSESVE
```

```
AtCsc1      286 ------------------------------QKVDAIEHYIAEIDKISKEI
CaPhm7      298 ------------------------------IKVDTLDYSPEKLGELNKEI
CaRsn1      285 ------------------------------SKVDTIRHCQEQIPILDKEV
CaSpo75     327 -----PIRIPQIERTIRITLPGWLRIFCFQKPIPMYEWSLQTLSECNQKI
C1_06270Wp  308 PLDKPQLNELANKYTQELIELDG-----------EIKHMQGKFDSDLSTI
C3_04720Cp  366 GDSSDTLNRLLNDESRTRPSLRKGWFGLFGPKVDSINYYTDKLEVIDKEI

AtCsc1      306 SKEREEVVNDPKAIMPAAFVSFKTRWAAAVCAQTQQTRNPTQWLTEWAP-
CaPhm7      318 TKEQT--EYQTYDQLPAVFIEFPSQLEMQKAYQAIPYQPDFKGVKTVINA
CaRsn1      305 KKLQKK--FRHSMPLNSIFVEFENQYYAQLAYQSTVHHNPMRMSPRFIGL
CaSpo75     372 DEEKLKLAEGQLAKHSKIFIEFTSQEGSYIAHQCLLSQSQGFLDKTTIEI
C1_06270Wp  347 DVKE----NREFKQVPSAFITMDSVASAQMAAQTILDPRVYKLIASLAP-
C3_04720Cp  416 TRAR----TREYPATSTAFLTMKTVAEAQMLAQAVLDPKVNHLITNLAP-
                                                    TM4  TM4
AtCsc1      355 EPRDVFWSNLAIPY-VSLTVRRLIMHVAFFFLTFFFIVPIAFVQSLATIE
CaPhm7      366 APEDIIWENLQLTS-MKRRIKSIIANTILTLLIIFWCIPVAVVGAISNIN
CaRsn1      353 EPKDVIHSNLRMFW-WERITRRFLAFAAIVALVVFWAIPVAAVGTISNIT
CaSpo75     422 NPNDIIWRNVCRNDGIACKFEKYLVTIAFISIIILYVIPVSLIGLVSQIP
C1_06270Wp  392 APKDIIWENLKLTY-FERKIKSYFITLVIVLSYGFIIFLVIPLTSLLDLK
C3_04720Cp  461 APHDIRWDNLSLTR-QDRNTKILAVTIFIGIMSLLLVYPVRFMASFLNTK
                                               TM5  TM5
AtCsc1      404 GIVKAAPFL-KFIVDDKFMKSVIQGFLPGIALKLFLAFLPSILMIMSKFE
CaPhm7      415 VLTDKVPFLKFILKMPDVIMGVITGLLPVVALTILMSLVPPFIKWMGKIS
CaRsn1      402 FLTNKLPWLRWILKMPHALLGLVTGLLPTILLSLLMFLLPIIIRVFARIS
CaSpo75     472 LLTQLLPFLEWIYQFPEEARETIAGFLPSILLGVLTEIVMIIFRFLTYFK
C1_06270Wp  441 TISKFWPALGEFIGQSKWLTTFVTGILPPLLFTLLNFSFPYFYQYLSQLQ
C3_04720Cp  510 SISKIWPSLGKAIESHKWAETLITGLLPTYLFTILNIVIPFFYVWISEKQ
                                       TM6  TM6
AtCsc1      453 GFTSISSLERRAAFRYYIFNLVNVFLASVIAGAAFEQLNSFLNQSANQIP
CaPhm7      465 GRLTIQQVESYCQSWYFAFQVVNVFLAIALGSSAAAVATQIVQNPGE-AL
CaRsn1      452 GEISAVGVEKWTQNAYFAFLMVNGFLVTALASSATATITEIIDKPTS-AM
CaSpo75     522 GRTTGCEVEIDLQKWYFAFLFVQQFLVVTISSSVTVILKQIIDQPTS-IP
C1_06270Wp  491 GYTSNSDVELSTLSKNFFFIFFNLFLIYVAAGTFWDYMSYISDTTK--IA
C3_04720Cp  560 GYLSHSDEELSSVSKNFFYIFVNLFLVFTTFG-----TASFVDTTK--IA
                                     TM7    TM7
AtCsc1      503 KTIGVAIPMKATFFITYIMVDGWAGVAGEILMLKPLIMFHLKN------A
CaPhm7      514 QKLSSSFPKSVNFYYSYLCLEGLTISSGVLLQIVALILSHILG------R
CaRsn1      501 SILANKLPLSSNFYISYLVLQGFSIAGGSLFQVVGLFLYYILG------T
CaSpo75     571 VLLATNLPKSATFFFQYISLRAFAFCGNNFLRISPLIQSLIVC------K
C1_06270Wp  539 VQLATSLRRMALFYVDLILLQGLTMFPVKLLQVSDFFLLNVLGKLFYFKR
C3_04720Cp  603 FDLARSLRDLSMFYVDLIILQGLGIFPFKLLLVGNLLRFLVN------SL
                                                TM8         TM8
AtCsc1      547 FLVKTDKDREE-AMDPGSIGFNTGEPRIQLYFLLGLVYAPVTPMLLPFIL
CaPhm7      558 ILDGTPRAKWTRWNTLGQPAYSTLYPGFQLLTVIALSYSVIAPLILGFTA
CaRsn1      545 LFDNTVRKKWNRFSGLGTVAWGTVFPIFTQLASITLAYSIISPLILVFAF
CaSpo75     615 FIDITPRQKFNRITNLPKIKWGTTFAVYSIYACIGISYSIISPLISIFII
C1_06270Wp  589 LILKTPRDYRSYYFTPQIFDFGINLPQHILIFMIILIYSVVSTKIVTCGL
C3_04720Cp  647 FRCKTPRDYLN-LYKPPVFNFGLQLPQPILIFIITLVYSVMSSKILTAGL
                     TM9                                 TM10
AtCsc1      596 VFFALAYIVYRHQIINVYNQE--YESAAAFWPDVHGRVIAALVISQLLLM
CaPhm7      608 IAFILFYFAYIYTMIFVLRPST-VDARGTNYVKSLFQLFTGLFLAQLWIT
CaRsn1      595 ASFFLVYVAYAHNITYCFVEG--PDAFGSHYPRALFQTFCGIYLGEIVLL
CaSpo75     665 FFLNLSILYYKYALKYVYSHINESETTGRLYPTALLHLYTGVYCLECCLI
C1_06270Wp  639 IYFILGLFVYKYQLVYNFVHP--PHSTGKVWPMIFRRVILGLIIFQLFMC
C3_04720Cp  696 LYFIIGYFVSKYQLLYACVHP--PHSTGKVWPIIFRRIILGLFLFQITMV
```

```
                                  TM11 TM9
AtCsc1       644 GLLGTKHAAL--------AAPFLIALPVLTIGFHHFCKGRYEPAFIRYPLQ
CaPhm7       657 AIFVFSKNWA--------CVALEGVIVVVTIAARLWMKWKFLPLVDAVPIS
CaRsn1       643 GIVVVGKGWG--------PIVIQVIVLLATIFAHVHLNLAFDHLLDVVPLD
CaSpo75      715 GVFFLSKNDKGGYPMRVQGWIMTGILILTIFANTIIYNRYIPHFSNLPIL
C1_06270Wp   687 GTLALESAIL--------LSILCTPLIFITMIILWNFEKYYVPLNTFIALR
C3_04720Cp   744 GTLALQDAIT--------CATFLAPLPFLTLYFWWSFHKQYIPLSTFIALR

AtCsc1       687 EAMMKDTLETAR---EP----------------------------------
CaPhm7       700 AIKYAAGDPTYSYPIHDQGLKEIKVEGKNYWEGGNQLGLGPDPKDQVLPD
CaRsn1       686 AMRAVDGVSSTASYTGPSEYESKVLNKKKR--------------R-----
CaSpo75      765 SDKTFKDG------------------------------------------
C1_06270Wp   730 AILNPYDFEKVFDQDDQSFSSTSNANSNDESD-------------TSAILD
C3_04720Cp   787 AIE-----------------------------------------------

AtCsc1       701 -------------------------NLNLKGYLQNAYVHPVF--KGDEDD
CaPhm7       750 RIPGNGPSSYAYDQQQQNHRGSDSSAVDTKVGHGESLDKPKSPFTDFNN
CaRsn1       717 ---ANFEKELLKEQEEESKLKADVKAQMESDYDQEEAIDTTS--VVPLLA
CaSpo75      773 ----------------------------AKPISESS----------SPN--
C1_06270Wp   768 DLDLDLDLENDPIDESSCLLENGGDNGNGNDHGSNGEINNGS--ITKSSR
C3_04720Cp   790 ---------------------------------SNENINPTD--LEQIIE

AtCsc1       724 YDIDDKLGK----------------------FED---------------E
CaPhm7       800 NHDAEKSAGFNPVNKAIAAPTQGVSWLTSFFQPKKDTFDIIKSDMPSSYF
CaRsn1       762 DRDFKTTESNNPIVRFIRPDVFLNFRYVKRILPA---------------T
CaSpo75      784 --TDDTCYSN----------------------------------------
C1_06270Wp   816 QNNGEPT------------------------ISR---------------R
C3_04720Cp   805 NNNNK---------------------------------------------

AtCsc1       737 AIIVPTKRQSRRNTPAPSIISGDDSPSLPFSGKLV---------------
CaPhm7       850 NYIEYHSDFIRHAYDDPAVTAEEPHIWIARDPMGLSEIEKNKALKEGVQV
CaRsn1       797 YNVEPKIVDNKHAYDAPAISAKCPGVWFPADPMGLSEIEI-EQLSPIVDI
CaSpo75      792 ---------HKLLYLHPAFKYESPKIWLPKDPHGYTDLSLQKMSDRSQFE
C1_06270Wp   827 KSTLDEEREEFTDYTYPGLIDPLDGPWVGFTGNFVKMIEY-KSLRLQQQA
C3_04720Cp   810 --TLDERRELNTKYEYPNLVNDLDGPMIALDGEDVLIVN-----------

AtCsc1       772 -------------------------------------------------
CaPhm7       900 SDENATFDDKG------------------ALIFTGPPPAYEEPIRV
CaRsn1       846 STKNATFNEKG------------------KIVFLGKPPN-------
CaSpo75      833 GENDGAQVQLG------------KFFSSLKLMISEAPPDYK-----
C1_06270Wp   876 LLEEGRNVRNENEAAIGTTFINEQELVVNKKLSVSEWE--------
C3_04720Cp   847 --RDGTTVRKP------------------PQYFSSEWDY-------
```

Figure S1. Amino acid sequence comparison. Transmembrane domain (TM) regions of AtCsc1 and CaPhm7 are underlined under their amino acid sequences, and TM names of AtCsc1 (**blue**) and CaPhm7 (**red**) are indicated above their amino acid sequences. TM domains were predicted with the online SMART software (http://smart.embl-heidelberg.de/). CaPhm7 shares 21%, 36%, 26%, 20% and 21% identities with AtCsc1, CaRsn1, CaSpo75, C1_06270Wp and C3_04720Cp, respectively, and shares 42%, 59%, 44%, 41% and 42% similarities with AtCsc1, CaRsn1, CaSpo75, C1_06270Wp and C3_04720Cp, respectively, in their amino acid sequences. Identical amino acid residues are indicated in yellow in color, and similar amino acid residues are indicated in light blue and green

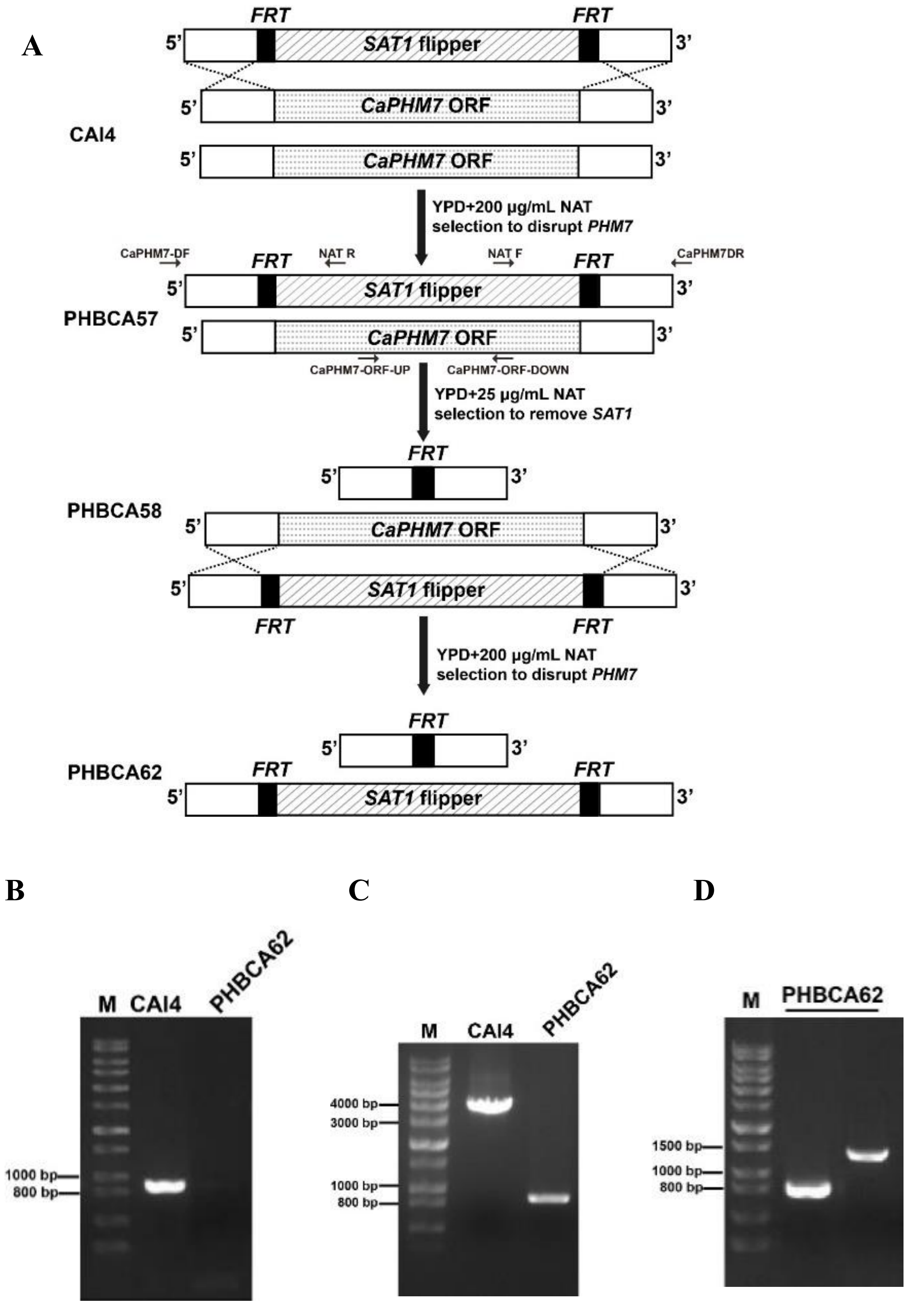

Figure S2. Disruption of the *Candida albicans CaPHM7* gene. (**A**) Disruption strategy of the two alleles. PCR confirmation of genotypes of the homozygous mutant PHBCA62 with primer pairs CaPHM7-ORF-UP/DOWN (**B**), CaPHM7-DF/DR (**C**) as well as CaPHM7-DF/NAT-R (middle lane) and CaPHM7-DR/NAT-F (last lane) (**D**).

```
CaPhm7     1  -MAGSSIPQSSNSSVSQFLSTLIPTLVVSVVFLLAFIVIHSKQRRVYEPR
CdPhm7     1  MADSSSDPQSSNSSVSQFISTLIPTLVISVVFLLGFIAIHSKQRRVYEPR
CgPhm7     1  ------MAADTSSSTSAFVTTLIFNGVVAVIFLLLFWYLKPKNKRVYEPR
CtPhm7     1  -MADDQNPQNTNSSVSQFLSTLIPTLVISVVFTLAFILVRNKRKRVYEPR
ScPhm7     1  --------MADSSSTSAFISTLIIYGLTAVVFVWLFLLLRPKNRRVYEPR
SpPhm7     1  ------MSDSSSSSTSAFVSSLVFNFAIFCAFIGLFLCLRPREKHVYQPR

CaPhm7    50  --AVVESLPDDLRTETAP-KGPFSWLTYLLAKPRTFYIQYAGTDGYFFLR
CdPhm7    51  --AVVESLPNDLRTETVP-KGPFNWLTYLLKKPRTFYIQFAGTDGYFFLR
CgPhm7    45  TLTDIQTISEEERIDTLEYDDENSWLGFLLSRPHSFLIQHCSIDGYLFLR
CtPhm7    50  --AVVKSLPQDLRPEPSP-GGLFSWLTTLLRKPSTFLIQFASTDGYFFLR
ScPhm7    43  SLKDIQTIPEEERTEPVP-EGYFGWVEYLLSKPHSFLIQHTSVDGYFLLR
SpPhm7    45  --CIIDTQPKEEKPEPSP-SSPFGLFAYVVKRSETYLIQYAGVDGYFFIR

CaPhm7    97  FLFEFFCVCVLGAVITWPILFPVNATNGNNNTPGSNVKGFDILTFANIKD
CdPhm7    98  FLFEFFCICVLGAVITWPILFPVNATNGNNNVPGSNVKGFDILTFANIKD
CgPhm7    95  YIGIFAGLSFISCFILFPILLPVNITNGNH------LEGFEVMSFANVRN
CtPhm7    97  FLFEFFCICVLGAIITWPILFPVNATNGKNNEPNSNVKGFDILTFSNIKD
ScPhm7    92  YIGIVGSLSFVGCLLLLPILLPVNATNGNN------LQGFELLSFSNVTN
SpPhm7    92  YLFTFGALCILGCLVLFPILLPVNATNGVGE------KGFDILSFSNVKN

CaPhm7   147  KWRTFAHVFLSWILFGAVIFLIYRELVYYVTYRHALQTTPLYDSLLSSRT
CdPhm7   148  KWRTFAHVFLSWLLFGAVIFLIYRELVYYVTYRHALQTTPLYDSLLSSRT
CgPhm7   139  KNRFYAHVFLSWIIFGLFTYVIYRELYFYISLRHSLQTTPLYDGLLSSRT
CtPhm7   147  KWRTFAHVFLSWLLFGAVIFLIYRELVYYVTYRHALQTTPLYDSLLSSRT
ScPhm7   136  KNRFYAHVFLSWIFFGLFTYVIYKELYYYVVFRHAMQTTPLYDGLLSSRT
SpPhm7   136  HNRFYAHVFLSWLFFGFTIFIIYRELRYYVIFRHAMQSSGLYNNLPSSST

CaPhm7   197  LLLTEISTEYLQDDK-LRGYFPTATNIWYSRDYKKLQKQVKERTKLANKY
CdPhm7   198  LLLTEISTEYLQDDK-LRGYFPTATNIWYSRDYKKLQKKVKERTKLANKY
CgPhm7   189  VVVTELSDTYNQEGE-FDRLFPNAAHIIFARNLKELQDMVKERDETAQNY
CtPhm7   197  LLLTEINTKYLDDEQ-LRTYFPTATNIWYARDYKKLEKTVKERTKLASKY
ScPhm7   186  VIVTELHKSIAQEGE-MQMRFPKASNVAFAYDLSDLQELCKERAKNAAKY
SpPhm7   186  MLLTELPNSVLNDEETLHELFPNASEFTCVRDLKKLEKKVKKRSDLGNKY

CaPhm7   246  EGTLNKVLTKAVKLRN-----KCLKKSKPAPEPQDDIDKYLKDGKKRP--
CdPhm7   247  EGTLNKVLTKAVKLRN-----KCLKKNKPAPEPQDDIDKYLKDGKKRP--
CgPhm7   238  EKTLNKLINKCVKKQN--SEKKREKLYKDG-KPKDDLSTYVPHNKRPKKW
CtPhm7   246  EGTLNKVLSKAVKLRN-----KCIKKGKPAPEPEDDIDKYLKDGKKRP--
ScPhm7   235  EAALNKVLNKCVKMTRNKTQKQLDKLYNNGTKPKDDLETYVPHKKRPK--
SpPhm7   236  ESTLNSLINKSVKKHN-----KLVKKHKPLP----STLDYTAYVKKRP--
```

```
CaPhm7   289 ---THKLKFLIGIKVDTLDYSPEKLGELNKEITKEQTEYQTYDQLPAVFI
CdPhm7   290 ---THRLKFLIGEKVDTLNYSPEKLNELNKEITKEQIEYQTYDQLPAVFI
CgPhm7   285 IKHWPLPTFLGGEKVDLLTYSTKQIGDLNDKIKDKQQDWQKSDHLNSVFL
CtPhm7   289 ---THKLKFLIGKKVDTLSYAPEKLGELNKDIAKQQAEYQTYEQLPAVFI
ScPhm7   283 HRLGKLPLCLGGKKVNTLSYSSKRIGELNEEIHEKQADWASNDRQPACFI
SpPhm7   275 ---THRLKFLIGKKVDTIDYCRDTIAELDEVVDKLQTSLEERKKVGSVFI

CaPhm7   336 EFPSQLEMQKAYQAIPYQPDFKGVKT---VINAAPEDIIWENLQLTSMKR
CdPhm7   337 EFPSQLELQKAYQAIPYQPDFKGVKT---VINAAPEDIIWENLQLTSMKR
CgPhm7   335 IFDTQLEAQRCFQSVPDILGFTNYGKC--LIGCTPDDLNWDNLNLTKKAR
CtPhm7   336 EFPSQLELQKAYQGIPYQPDFKGVKT---VINAAPEDIIWENLQLTPVKR
ScPhm7   333 QFETQLEAQRCYQSVEAILGKKNFGKR--LIGYSPEDVNWGSMRLSSKER
SpPhm7   322 RFRSQTDLQTAYQAFLYSKKFRKYRFGRALVGIAPEDIVWSNLDLSMYTR

CaPhm7   383 RIKSIIANTILTLLIIFWCIPVAVVGAISNINVLTDKVPFLKFILKMPDV
CdPhm7   384 RIKSIIANTILTLLIIFWCIPVAVVGSISNINVLTDKVPFLKFILNMPDV
CgPhm7   383 YIKRLTANSILTAMIIFWAIPVAVVGCISNVNFLVEKIHFLHFLNNVPNV
CtPhm7   383 IIKKIIANTILTLMIIFWCIPVAVVGAISNINVLTDKVHFLRFILNMPKV
ScPhm7   381 HSRRAVANTIMVLLIIFWAFPVAVVGIISNVNFLTDKVPFLRFINNMPTF
SpPhm7   372 RGKKTISNTILTLMIIFWAFPVAVVGCISNVNYLIEKVHFLKFIDHMPPK

CaPhm7   433 IMGVITGLLPVVALAILMSLVPPFIKWMGKISGRLTIQQVESYCQSWYFA
CdPhm7   434 IMGVITGLLPVVALAILMSLVPPFIKWMGKISGRLTIQQVESYCQSWYFA
CgPhm7   433 IMGIITGLVPSLALSILMSLVAPFIKKIGEMSGDITRQETDQYCQKWYFA
CtPhm7   433 IMGVITGLLPVVALAILMSLVPPFIKWMGKLSGRLTVQQVESYCQSWFFA
ScPhm7   431 LMGVITGLLPTIALVVLMSLVPPFIVMLGKLSGCVTRQETDLYSQAWYYA
SpPhm7   422 LLGIITGILPSVALSILMSLVPPFIKFLGKFGGALTVQEIENYCQNWYYA

CaPhm7   483 FQVVNVFLAIALGSSAAAVATQIVQNPGEALQKLSSSFPKSVNFYYSYLC
CdPhm7   484 FQVVNVFLAIALGSSAAAVATQIVQDPGAALKKLSSSFPKSVNFYYSYLC
CgPhm7   483 FQVLNTFIVTTLASSASSTVTAIIDEPGSAMTLLANNLPKASNFFITYFL
CtPhm7   483 FQVVNVFLAMALGSSAAAVATQIVEDPGKALQQLSSNFPKSVNFYYSYLC
ScPhm7   481 FTVIQIFLVVTATSSASSTVDSIIDRPRSAMTLLANNLPKASNFYIMYFI
SpPhm7   472 FQVVQVFLVTTMTSAATSAVVQVIKEPASSMTLLASNLPKASNFYISYFL

CaPhm7   533 LEGLTISSGVLLQIVALILSHILGRILDGTPRAKWTRWNTLGQPAYSTLY
CdPhm7   534 LQGLTISSGVLLQIVALILSHILGRILDGTPRAKWTRWNTLGQPAYSTLY
CgPhm7   533 LQGLTMPTGQLLQVANLILSKFMGRILDTTPRQKWNRYNTLSKPSMGVVY
CtPhm7   533 LQGLTISSGTLLQLVALILSHILGRILDGTPRAKWNRWNTLGQPAYSTLY
ScPhm7   531 LKGLTGPTWTILQAVNLLLSKVLGRVLDSTPRQKWNRYNTLATPRMGIVY
SpPhm7   522 LQGLSIPGGALLQIVTLLLSKVLGRIFDNTPRKKWNRWNQLSAPSWGTVY
```

```
CaPhm7   583  PGFQLLTVIALSYSVIAPLILGFTAIAFILFYFAYIYTMIFVLRPSTVDA
CdPhm7   584  PGFQLLTVVALSYSVIAPLILGFTAIAFILFYFAYIYTMIFVLRPSTVDA
CgPhm7   583  PTVEILVCIMISYIIIAPILLVFSTMTFLFLYFAYLYNLNFVMG-FSFDL
CtPhm7   583  PGFQLLTVIALAYSVIAPLILGFTAIAFILFYFAYIYTFVYVLRPSTVDA
ScPhm7   581  PGIEILVCIYICYSIIAPILLFFSTVMLTLLYVAYLYNLNYVFG-FSFDL
SpPhm7   572  PVYSLLVTIMICYSIIAPIIIGFAAVAFVLIYFAYSYNLIYVLG-HNADA

CaPhm7   633  RGTNYVKSLFQLFTGLFLAQLWITAIFVFSKNWACVALEGVIVVVTIAAR
CdPhm7   634  RGTNYVKSLFQLFTGLFLAQLWITAIFVFSKNWACVALEGVIVVVTIASW
CgPhm7   632  KGRNYPRALFQVFVGIYLSEVCLLGLFIMAKAWGPLVLECFWIVVTALAH
CtPhm7   633  RGTNYVKSMFQLFTGLYLAQLWITAIFVFSKNWACVVLEAVIVVVTFVSH
ScPhm7   630  KGRNYPRALFQIFVGIYLSEVCLLGLFIMAKTWGPLVLEVFWIVVTALAH
SpPhm7   621  KGRNYPRALFQVFVGLYLAEVCLIGLFVLAKNWGATVLEAVFLGFTVACH

CaPhm7   683  LWMKWKFLPLVDAVPISAIKYAAGDPTYSYPIHDQGLKEIKVEGKNYWEG
CdPhm7   684  IWMKWKFLPLVDAVPISAIKYAAGDPTYSYPMHDQGSKEIKLEGENYWEG
CgPhm7   682  IYMKWRFLPLIDAIPLSAILNARGSKKHRYPQKDQGLKEVKGIGEDMKKL
CtPhm7   683  MWMKRRFLPLIDAVPISAIRYAAGDSSSAYPMEDQGYREIKREGENYWEG
ScPhm7   680  IYMKRKFIPLFDAVPLSAIRHARGEPGYSYPTSDLGLQEIKDIADEMKGK
SpPhm7   671  LYFKYKFLPLMDAVPISAIESVSERPEIKYPMDL-GTSEMKNVGRAYPEI

CaPhm7   733  GNQLGLGPDPKDQVLPDRIPGNGPSSY--AYDQQ----------------
CdPhm7   734  GNQLGLGPDPKDQVLPDKIPGNSTSSYGHGYDQQ----------------
CgPhm7   732  FEDDDHNGVLRPATKGDLRRADLLPEDELESDS-------EGCGDDISTL
CtPhm7   733  GNQLGLTQEPKDQVLPSRIP----QS---NIDHV----------------
ScPhm7   730  YEQDNTHGILTPVTKDDLKKANLIPDNDGSSENGTPSNPFESGSERASLS
SpPhm7   720  LEKLSSS-SGSDEFLETSSR---------TSEN-----------------

CaPhm7   765  ---------------QQQN----HRGSDSSAVDTKVGHGESVDKPKSPFT
CdPhm7   768  ---------------QQQQQQQYHRGSDSSAVDTKVGHGESVDKPKSPFT
CgPhm7   775  G-----DMEESLPKNDRKQS---NVTDGSKYASTFVGSDEDFKKLHYADV
CtPhm7   760  ---------------QPGR-----RESDSSAIDTKVGHGESFEKAHSPFS
ScPhm7   780  GSNAEGDSIKKLNDTVIKKSSTLSPSTKDNNESTFVPEGEKFRKFHYSDV
SpPhm7   743  -------------------------------------TKEKIDKDDEGFA

CaPhm7   796  DSNN----------------------------NHDAEKSAGFN-PVNKAI
CdPhm7   803  DSNNKD--------------------------NHDPEKSAGFN-PVNKAV
CgPhm7   817  KDMKKQ----QQSGPEVNPEGAVVGNADVGKIFSDPMAMTDDPNAFPDNI
CtPhm7   790  DANR-----------------------------HDPEKSTGIVNPINAAV
ScPhm7   830  EALRNKRPYDEDDHSKHGPEGAVPVNADAGVIYSDPAAVMKEPQAFPPDV
SpPhm7   756  ITNIS-----------------------------SVHKMPSFVLSYFSDL
```

```
CaPhm7   817 AAPTQGVSWLTSFFQP-KKDTFDIIKSDMPSSYFNYIEYHSDFIRHAYDD
CdPhm7   826 AAPTQGVSWLTTFFQP-KKDTFDLIRSDMPNSYFNYIEYHSDFIRHAYDD
CgPhm7   863 LPSQGKFKRLANFFEPSKSYPFCKVRNRLPHSLNTTIQYNLDYAETAYTD
CtPhm7   811 SAPGKGVSWLTRFFQP-KKETFDMIKSILPDCYYNYIEYNEEFIRHAYDD
ScPhm7   880 LETNTWTRRILQFFNPRRSYPFDSVRMRFPLVFNTSIEYDEEYLSSAYTD
SpPhm7   777 AASNRILTGFDRVLQL-LPSFYDIP---------VRVR--N----VQYVS

CaPhm7   866 PAVTAEEPHIWIARDPMGLSEIEKNKALKEGVQVSDENATFDDKGALIFT
CdPhm7   875 PAVVAEEPHIWIARDSMGLSEIEKNKALKEGVQVSDENATFDDKGALIFT
CgPhm7   913 PSVNSTSPIVWICRDPMGVSQRQIDEAHMDNVPITDEFTEYDEKGGAQFT
CtPhm7   860 PAVTDEEPHIWVAKDPMGLSEIEKNKALKEGVDVSDENATFDDKGQLIFT
ScPhm7   930 PCVREKDPIVWCCKDPLGVSKQQIQEARSNGLDVRDDFTRYDEKGKVIFT
SpPhm7   811 PALKATPPSVWIPKDPLGLSTYAIEDARGK-VDIFDDNTTFNEKGNLQYT

CaPhm7   916 GPPPAYEEPIRV-
CdPhm7   925 GPPPAYEEPIRV-
CgPhm7   963 FNPPDFIRKAKK-
CtPhm7   910 GPPPSYEEAIRV-
ScPhm7   980 YNPPDYEPEAKK-
SpPhm7   860 GPPPDYDEAIRS-
```

Figure S3. Transmembrane domains (TMs) of AtCsc1, ScPhm7 and CaPhm7 as well as the amino acid sequence comparison of *Candida albicans* CaPhm7 with its homologs. CaPhm7 shares 94% (98%), 42% (64%), 82% (91%), 43% (64%) and 46% (67%) identity (similarity), respectively, with *C. dubfiniensis* CdPhm7, *C. glabrata* CgPhm7, *C. tropicalis* CtPhm7, *Saccharomyces cerevisiae* ScPhm7 and *Schizosaccharomyces pombe* SpPhm7. Identical amino acid residues are indicated in yellow in color, and similar amino acid residues are indicated in light blue and green.

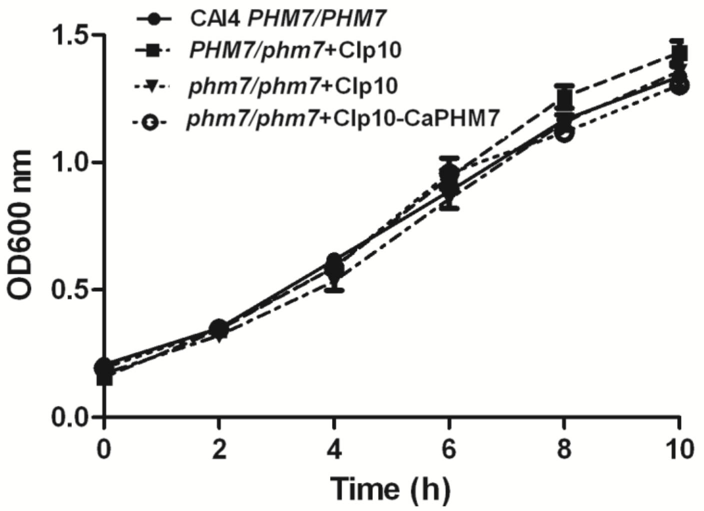

Figure S4. Growth assay of the wild type CAI4+CIp10, the heterozygous mutant *PHM71/phm7*+CIp10, the homozygous mutant *phm71/phm7*+CIp10 and the revertant *phm7/phm7*+CIp10-CaPHM7. Cells were cultured in SD-URA medium at 30ºC overnight, diluted for 10 times in fresh YPD medium and grown further for indicated hours. Data were the average of three independent experiments. Growth rates of the heterozygous mutant, the homozygous mutant and the revertant were not significantly different from the wild type.

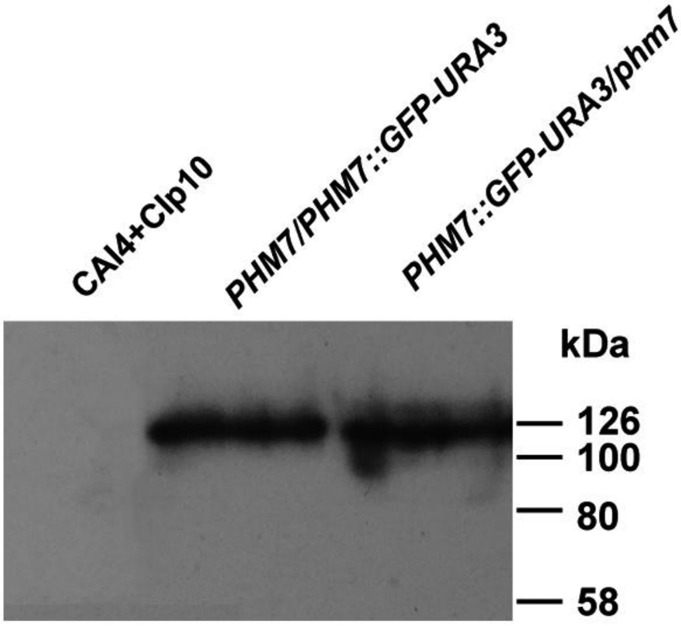

Figure S5. Western blot analysis. The CAI4 (XDCA06) control, PHBCA107 and PHBCA126 cells were grown to log-phase in SD-URA medium and collected for total protein extraction. Western blot analysis was carried out with the monoclonal anti-GFP antibody. The CaPHM7-GFP protein of 126 kDa was indicated.

```
CaPhm7     1 MAGSSIPQSSNSSVSQFLSTLIPTLVVSVVFLLAFIVIHSKQRRVYEPRA
HsAno10    1 ------MKVTLSALDTSESSFTPLVVIELAQDVKEETKEWLKNRIIAKKK
ScIst2     1 ------MSQTITSLDPNCVIVFNKTSSANEKSLNVEFKRLNIHSIIEPGH

CaPhm7    51 VVESLPDDLRTETAPKGP-FSWLTYLLAKPRTFYIQYAGTDGYFFLRFLF
HsAno10   45 DGG--------------------AQLLFRPLLNKYEQETLE-----NQNL
ScIst2    45 DLQTSYAFIRIHQDNAKPLFSFLQNLDFIESIIPYHDTELS-----DDLH

CaPhm7   100 EFFCVCVLGAVITWPILFPVNATNGNNNTPGSNVKGFDILTFANIKDKWR
HsAno10   70 YLVGASKIRMLLGAEAVGLVKECNDN------------------------
ScIst2    90 KLISISKSKILEAPKQYELYNLSNLTNNPKQSLYFAFLQNYIKWLIPFSF

CaPhm7   150 TFAHVFLSWILFGAVIFLIYRELVYYVTYRHALQTTPLYDSLLSSRTLLL
HsAno10   96 --------------------------TMRAFTYRTRQNFKGFDDNNDDFL
ScIst2   140 FGLSIRFLSNFTYEFNSTYSLFAILWTLSFTAFWLYKYEPFWSDRLSKYS

CaPhm7   200 TEISTEYLQDDKLRGYFPTATNIWYSRDYKKLQKQVKERTKLANKYEGTL
HsAno10  120 TMAECQFIIKHELENLR--------AKDEKMIPG---YPQAKLYPGKSLL
ScIst2   190 SFSTIEFLQDKQKAQKK--------ASSVIMLKKCCFIPVALLFGAILLS

CaPhm7   250 NKVLTKAVKLRNKCLKKSKPAPEPQ---DDIDKYLKDGKKRPTHKLKFLI
HsAno10  159 RRLLTSGIVIQVFPLHD------------------SEALKKLEDTWYTRF
ScIst2   232 FQLYCFALEIFIKQIYNGPMISILSFLPTILICTFTPVLTVIYNKYFVEP

CaPhm7   297 GIKVDTLDYSPEKLGELNKEITKEQTEYQTYDQLPAVFIEFPSQLEMQKA
HsAno10  191 ALKYQPIDSIR--------------GYFGETIALYFGFLEYFTFALIPMA
ScIst2   282 MTKWENHSSVVN-AKKSKEAKNFVIIFLSSYVPLLITLFLYLPMGHLLTA

CaPhm7   347 YQAIPYQP---------------------------DFKGVKTVINAAPED
HsAno10  227 VIGLPYY--------------------------LFVWEDYDKYVIFASFN
ScIst2   331 EIRTKVFNAFSILARLPTHDSDFIIDTKRYEDQFFYFIVINQLIQFSMEN

CaPhm7   370 IIWENLQLTSMKRRIKS------------IIANTILTLLIIFWCIPVAVV
HsAno10  251 LIWSTVILELWKRGCAN------------MTYRWGTLLMKRKFEEPRPGF
ScIst2   381 FVPSLVSIAQQKINGPNPNFVKAESEIGKAQLSSSDMKIWSKVKSYQTDP

CaPhm7   408 GAISNINVLTDKVPFLKFILKMPDVIMGVITGLLPVVALAILMSLVPPFI
HsAno10  289 HGVLGINSITGKE---EPLYPSYKRQLRIYLVSLPFVCLCLYFSLY----
ScIst2   431 WGATFDLDANFKK---LLLQFGYLVMFSTIWPLAPFICLIVNLIVYQ---

CaPhm7   458 KWMGKISGRLTIQQVESYCQSWYFAFQVVNVFLAIALGSSAAAVATQIVQ
HsAno10  332 ------------------VMMIYFDMEVWALGLHENSGSEWTSVLLYVPS
ScIst2   475 --------------VDLRKAVLYSKPEYFPFPIYDKPSS--VSNTQKLTV

CaPhm7   508 NPGEALQKLSSSFPKSVNFYYSYLCLEGLTISSGVLLQIVALILSHILGR
HsAno10  364 IIYAIVIEIMNRLYRYAAEFLTSWENHRLESAYQNHLILKVLVFNFLN--
ScIst2   509 GLWNSVLVMFSILGCVITATLTYMYQSCNIPGVGAHTSIHTNKAWYLA--

CaPhm7   558 ILDGTPRAKWTRWNTLGQPAYSTLYPGFQLLTVIALSYSVIAPLILGFTA
HsAno10  412 ------------------CFASLFYIAFVLKDMKLLRQSLATLLITSQIL
ScIst2   557 ------------------NPINHSWINIVLYAVFIEHVSVAIFFLFSSIL

CaPhm7   608 IAFILFYFAYIYTMIFVLRPSTVDARGTNYVKSLFQLFTGLFLAQLWITA
HsAno10  444 NQIMESFLPYWLQRKHGVRVKRKVQA--LKADIDATLYEQVILEKEMGTY
ScIst2   589 KSSHDDVANGIVP-KHVVNVQNPPKQ--EVFEKIPSPEFNSNNEKELVQR
```

```
  CaPhm7   658 IFVFSKNWACVALEGVIVVVTIAARLWMKWKFLPLVDAVPISAIKYAAG-
 HsAno10   492 LGTFDDYLELFLQFGYVSLFSCVYPLAAAFAVLNNFTEVNSDALKMCR--
   ScIst2  636 KGSANEKLHQELGEKQPASSANGYEAHAATHANNDPSSLSSASSPSLSSS

  CaPhm7   707 ---------DPTYSYPIHDQGLKEIKVEGKNYWEGGNQLGLGPDPKDQVL
 HsAno10   540 -------VFKRPFSEPSANIGVWQLAFETMSVI-------------SVVT
   ScIst2  686 SSSSKTGVVKAVDNDTAGSAGKKPLATESTEKR-------------NSLV

  CaPhm7   748 PDRIPGNGPSSYAYDQQQQQNHRGSDSSAVDTKVGHGESVDKPKSPFTDS
 HsAno10   570 NCALIGMSPQVNAVFPE---------------------------------
   ScIst2  723 KVPTVGSYGVAGATLPETIPTSKNYYLRFDEDGKSIRDAKSSAES--SNA

  CaPhm7   798 NNNHDAEKSAGFNPVNKAIAAPTQGVSWLTSFFQPKKDTFDIIKSDMPSS
 HsAno10   587 -------SKADLILIVVAVEHALLALKFILAFAIP---------------
   ScIst2  771 TNNNTLGTESKLLPDGDAVDALSRKIDQIPKIAVTG--GENNENTQAKDD

  CaPhm7   848 YFNYIEYHSDFIRHAYDDPAVTAEEPHIWIARDPMGLSEIEKNKALKEGV
 HsAno10   615 ---------DKPRHIQMKLARLEFESLEALKQQQMKLVTENLK---EEPM
   ScIst2  819 AATKTPLIKDANIKPVVNAAVNDNQSKVSVATEQTKKTEVSTK---NGPS

  CaPhm7   898 QVSDENATFDDKGALIFTGPPPAYEEPIRV--------------------
 HsAno10   653 ESGKEKAT------------------------------------------
   ScIst2  866 RSISTKETKDSARPSNNNTTTTTTTDATQPHHHHHHHRHRDAGVKNVTNN

  CaPhm7   928 --------------------------------
 HsAno10   661 --------------------------------
   ScIst2  916 SKTTESSSSSSAAKEKPKHKKGLLHKLKKKL-
```

Figure S6. Amino acid sequence comparison of *C. albicans* CaPhm7, *S. cerevisiae* ScIst2 and human HsAno10. CaPhm7 shares 18% (34%) and 14% (30%) identity (similarity) with HsAno10 and ScPhm7, respectively, in their amino acid sequences. Identical amino acid residues are indicated in yellow in color, and similar amino acid residues are indicated in light blue and green

Table S1. Primers used in this study

Primer name	Sequence (5’to 3’)	Restriction site
CaPHM7-DF	gggagggattcttattcttgagt	
CaPHM7-DR	gtcgaatcagaacctacacctca	
CaPHM7-ORF-UP	cagtagctgttgttggtgcc	
CaPHM7-ORF-DOWN	ggaacagcatcgactaatgg	
CaPHM7-NAT-UP	atttgctttcctagcttattcatttcatcttaactaattaattggattaattgt<br>tatcatttatttttattaatcaagtttttttattaagaacagacaaattaac<br>cctcactaaaggg	
CaPHM7-NAT-DOWN	caaaaaaaataccatttaaatatacaaaaatcaaaaaaaaaaccaca<br>acgaacaatgatctgttttgtcttctctttttctttttttattataattattaaa<br>tacgactcactatаggg	
NAT-F	gctggagcattaacattggt	
NAT-R	gtgccgtgcaagtttctatc	
CaPHM7-GFP-UP	tcaagtttctgatgaaaatgccacttttgatgataaaggtgctctcatct<br>ttactggtcctccaccagcttatgaagaaccaattagagtaggtggtg<br>gttctaaaggtgaagaattatt	
CaPHM7-GFP-DOWN	ttaaaacattaaaataaacaagaataagtaataacgataaaaaaaaagt<br>tatgataaaactaaactatgataacaatttaagaatccaaaaaaaata<br>ctctagaaggaccacctttgattg	
CaPHM7-GFP-F	gctgaagaaccacatatttg	
CaPHM7-GFP-R	cgaatcagaacctacacctc	
GFP-R	ccatgtgtaatcccagcag	
CaURA3-F	gatatgggtggacaagaagaag	
pRS316-CaPHM7-F	gtcgacggtatcgat<u>aagctt</u>gtaattcattggatcattgttgtgtca	*Hind*III
CaPHM7TY-UP-R	gccaagcttgcatgc<u>ctgcag</u>ctgatgaacccaagg	*Pst*I
CaPHM7TY-DOWN-F	ccttgggttcatcag<u>ctgcag</u>	*Pst*I
CaPHM7TY-DOWN-R	gccaagcttgcatgc<u>ctgcag</u>ggtggtaatcccaactagaaagga	*Pst*I
M13F	tgtaaaacgacggccagt	
M13R	caggaaacagctatgacc	
CIp10-R	gacctacaccgaactgagatac	
CaRPS1-R	gccataaatatgccgattctc	
CIp10-URA3-F	gaaatgctggttggaatgct	
CaRPS1-F	gtcgtctgaatgtcgcaac	
